# Improved Prognostic Stratification with the FIGO 2023 Staging System in Endometrial Cancer: Real-World Validation in 2969 Patients

**DOI:** 10.3390/cancers17172871

**Published:** 2025-09-01

**Authors:** Jun-Hyeong Seo, Soo-Min Kim, Yoo-Young Lee, Tae-Joong Kim, Jeong-Won Lee, Byoung-Gie Kim, Chel Hun Choi

**Affiliations:** 1 Gynecologic Cancer Center, Department of Obstetrics and Gynecology, Samsung Medical Center, Sungkyunkwan University School of Medicine, Seoul 06351, Republic of Koreayooyoung.lee@samsung.com (Y.-Y.L.); tj28.kim@samsung.com (T.-J.K.); garden.lee@samsung.com (J.-W.L.); bksong.kim@samsung.com (B.-G.K.); 2 Department of Obstetrics and Gynecology, Samsung Medical Center, Sungkyunkwan University School of Medicine, Seoul 06351, Republic of Korea; rlatnals1130@naver.com

**Keywords:** endometrial cancer, FIGO 2023 staging, stage migration, survival analysis, prognostic performance

## Abstract

Background/Objectives: To assess the impact of the 2023 FIGO staging revision on stage distribution, survival outcomes, and prognostic performance in endometrial cancer compared to the 2009 system. Methods: This retrospective cohort study analyzed 2969 patients with FIGO 2009 stage I–III endometrial cancer diagnosed at Samsung Medical Center (1994–2023). Patients were reclassified per the 2023 FIGO system. Stage migration, progression-free survival (PFS), and overall survival (OS) were evaluated. Prognostic performance was compared using the Akaike information criterion (AIC), Bayesian information criterion (BIC), concordance index (C-index), and area under the receiver operating characteristic curve (AUC). Results: Stage migration occurred in 20.2% of patients, with 98.3% involving upstaging from FIGO 2009 stage I, largely due to the inclusion of aggressive histology, p53 abnormality, and substantial lymphovascular space invasion (LVSI). The proportion of stage I tumors decreased from 81.5% to 65.2%, while stage II increased to 21.9%, including 14.8% newly classified as stage IIC. Patients remaining in stage I showed favorable outcomes (5-year PFS: 95.3%, OS: 98.5%), whereas those upstaged—especially to stage IIC—had significantly worse outcomes (5-year PFS: 76.5%, OS: 83.1%). Tumors with p53 abnormalities had poorer survival (PFS: 70.8%, OS: 76.6%). The 2023 FIGO system outperformed the 2009 system in prognostic discrimination across all metrics. Conclusions: The FIGO 2023 staging revision improves prognostic accuracy in endometrial cancer by integrating histopathologic and molecular risk factors. These refinements enhance risk stratification and may support more individualized treatment strategies.

## Simple Summary

Endometrial cancer is one of the most prevalent gynecologic malignancies, with the majority of cases diagnosed at an early stage. Accurate staging is critical for prognostication and treatment planning. In 2023, the International Federation of Gynecology and Obstetrics (FIGO) introduced a revised staging system that integrates histopathologic and molecular features alongside anatomic parameters. While conceptually promising, its prognostic relevance has been supported by retrospective analyses. Nevertheless, broader validation across large, diverse real-world populations remains limited. In this study, we retrospectively reclassified nearly 3000 patients initially staged under the 2009 criteria to evaluate the impact of the 2023 revision on stage distribution and survival outcomes. Our findings revealed substantial stage migration and improved discrimination of patient prognosis, particularly in early-stage disease. The updated system more effectively stratified risk and demonstrated superior performance in survival prediction metrics. These results support the clinical applicability of the 2023 FIGO staging system and suggest that its implementation may enhance individualized risk assessment and inform more precise treatment strategies.

## 1. Introduction

Endometrial cancer (EC) ranks as the sixth most common malignancy among women globally, with its incidence steadily rising over the past decade. In South Korea, the incidence increased annually by 8.7% between 2014 and 2017, highlighting a growing healthcare burden [1,2]. Historically, the 2009 staging system introduced by the International Federation of Gynecology and Obstetrics (FIGO) has provided the foundation for classifying EC, guiding both surgical staging and treatment decisions based on anatomical spread [3]. However, evolving insights from pathology and molecular oncology have increasingly exposed the limitations of a system grounded solely in anatomical criteria, underscoring the need for a biologically informed staging framework [4,5,6].

In response, FIGO revised the staging system in 2023 to incorporate tumor histology and molecular features alongside conventional pathological parameters [7]. Major revisions include distinguishing tumors as aggressive or non-aggressive subtypes, assigning substantial lymphovascular space invasion (LVSI) cases to stage IIB, and permitting downstaging for selected ovarian metastases. Molecular classification, informed by The Cancer Genome Atlas (TCGA) model, now plays a pivotal role, with POLE-mutant tumors categorized as stage IA and p53-abnormal tumors reclassified as stage IIC. These updates primarily affect early-stage disease, which constitutes nearly 80% of newly diagnosed EC cases [8].

Despite these conceptual improvements, the real-world clinical impact of the 2023 FIGO system remains unclear. The prognostic utility of integrating histopathological and molecular features has yet to be fully validated, particularly regarding its influence on treatment strategies for early-stage disease. This study seeks to evaluate the effects of the 2023 FIGO revisions on stage distribution and survival outcomes among patients originally classified as stage IIIC or lower under the 2009 system. By analyzing patterns of stage migration and comparing survival outcomes, we aim to determine whether the updated classification improves prognostic accuracy and better informs therapeutic decision-making.

## 2. Materials and Methods

### 2.1. Study Design and Population

This retrospective cohort study was conducted at Samsung Medical Center, South Korea, and included patients diagnosed with endometrial cancer who received initial treatment between 1 September 1994 and 31 May 2023. Data collection and reporting adhered to the Strengthening the Reporting of Observational Studies in Epidemiology (STROBE) guidelines to ensure methodological rigor [9]. Eligible patients had histologically confirmed endometrial cancer, completed primary treatment (surgery and/or adjuvant therapy) at the institution, and had comprehensive clinical, pathological, and follow-up data, including recurrence and survival outcomes. Patients were excluded if they underwent partial or complete treatment elsewhere, had insufficient information to determine FIGO 2009 or 2023 stage even after retrospective chart review, received fertility-preserving treatments, or had uncertain histopathological diagnoses. Additionally, patients with FIGO 2009 stage IV disease were not included in the final analysis.

### 2.2. Treatment and Follow-Up

Surgical management was determined according to disease stage. Minimally invasive surgery (MIS), such as laparoscopy and robotic-assisted surgery, were preferred for early-stage disease, whereas open laparotomy was reserved for advanced-stage cases requiring cytoreductive or palliative interventions. Postoperative adjuvant therapy was administered based on guidelines from the European Society of Gynecological Oncology (ESGO), European Society for Radiotherapy and Oncology (ESTRO), and European Society of Pathology (ESP) [10]. Platinum-based chemotherapy, external beam radiation therapy (EBRT), and/or vaginal brachytherapy were selected according to individual patient risk factors, histological subtypes, and molecular profiles. Lymphadenectomy was not uniformly performed across all patients. The decision to perform lymph node staging was individualized, depending on disease stage, histologic subtype, patient comorbidities, and the treating surgeon’s judgment. Follow-up assessments included regular clinical examinations, transvaginal ultrasound, computed tomography (CT), and/or magnetic resonance imaging (MRI), with recurrence and survival outcomes systematically recorded in institutional cancer registries.

Across the study period from 1994 to 2023, the availability of diagnostic imaging and surgical techniques evolved, including the adoption of minimally invasive surgery. Radiotherapy modalities and adjuvant treatment recommendations also changed in step with contemporaneous institutional protocols and international guidelines. Patient management at each time point reflected the prevailing standards of care.

### 2.3. Staging and Data Collection

Patients were retrospectively staged according to both the 2009 and 2023 FIGO criteria to evaluate patterns of upstaging and downstaging. The 2023 system incorporated histological grade, LVSI, and molecular features into stage assignment. Tumors were classified as non-aggressive (grade 1–2 endometrioid carcinomas) or aggressive (grade 3 endometrioid, serous, clear cell, carcinosarcoma, or rare histology). The presence of ovarian metastases was re-evaluated according to new downstaging criteria. Molecular classifications, including POLE-mutant, mismatch repair (MMR)-deficient, p53-abnormal, and no specific molecular profile (NSMP) categories, informed additional stage reassignments, particularly among stage II and lower-stage tumors.

Clinical and pathological data were extracted from electronic medical records and institutional databases. Variables collected included demographics (age, body mass index (BMI), year of diagnosis), tumor characteristics (histologic subtype, grade, depth of myometrial invasion, cervical stromal involvement, LVSI status, peritoneal metastasis), and molecular profile. LVSI status was routinely assessed during pathological examination. Negative cases were reported as such, and positive cases were further classified as focal or substantial according to standard criteria [5]. Molecular testing was not uniformly performed for all patients but was selectively undertaken at the discretion of pathologists or treating physicians. POLE mutation status was determined by next-generation sequencing (NGS) when available. p53 status was assessed by immunohistochemistry on formalin-fixed, paraffin-embedded tumor sections. Aberrant expression was defined as diffuse strong nuclear overexpression, complete absence of staining (null pattern), or cytoplasmic staining. These cases were categorized as p53-abnormal, and in our dataset were denoted as p53-mutated. All other cases were considered wild-type. MMR status was evaluated by immunohistochemistry for MLH1, PMS2, MSH2, and MSH6, with loss of nuclear expression of one or more proteins interpreted as MMR-deficient and retained nuclear expression of all four proteins interpreted as MMR-proficient. Data regarding initial treatment modality and adjuvant therapies were also collected.

### 2.4. Outcomes and Statistical Analysis

The primary endpoints were 5-year progression free survival (PFS) and overall survival (OS), assessed according to both the 2009 and 2023 FIGO staging systems. Secondary endpoints included an evaluation of stage migration trends and the comparative prognostic performance between the two staging systems. In addition, exploratory subgroup analyses were performed according to molecular features (MMR-deficient vs. MMR-proficient and p53-abnormal vs. wild-type), given their integration into the 2023 FIGO classification and their known prognostic relevance in endometrial cancer [11,12]. PFS was defined as the interval from the date of first treatment to the date of documented recurrence or progression, and OS was defined as the interval from the date of first treatment to death from any cause or last follow-up. Patients without recurrence or death at last contact were censored. Survival distributions were estimated using the Kaplan–Meier method, and differences between groups were compared using the log-rank test. Cox proportional hazards regression models were applied to estimate hazard ratios (HRs) with 95% confidence intervals (CIs).

To evaluate the prognostic performance of the 2009 versus 2023 FIGO staging systems, model fit was compared using the Akaike information criterion (AIC) and Bayesian information criterion (BIC). Discriminatory ability was assessed using Harrell’s concordance index (C-index), and predictive accuracy was quantified by the receiver operating characteristic (ROC) curve analysis, with the area under the curve (AUC) calculated for 5-year PFS and OS.

All statistical tests were two-sided, and a *p*-value < 0.05 was considered statistically significant. All statistical analyses were performed using R software version 4.2.3 (R Foundation for Statistical Computing, Vienna, Austria).

## 3. Results

### 3.1. Study Population

Of the 3992 patients treated for endometrial cancer at our institution, 2969 patients diagnosed with FIGO 2009 stage I–III disease were eligible for the final analysis (Figure S1). The mean age at diagnosis was 53.7 years, and the mean BMI was 25.0 kg/m^2^. Endometrioid histology accounted for the majority of cases (88.5%), with low-grade tumors comprising 79.0% of the cohort. Myometrial invasion confined to the inner half was observed in 75.3% of patients, while cervical stromal invasion and substantial LVSI were present in 12.4% and 12.8% of cases, respectively. MIS was employed in 66.8% of patients, and most patients (59.8%) did not require adjuvant therapy. Molecular profiling data were available for 552 patients (18.6%), with p53 status assessed in 506 cases and MMR status evaluated in 230 cases. Routine POLE mutation testing was not performed in this cohort; therefore, the exact proportions of POLE-mutant and NSMP tumors could not be determined (Table 1).

### 3.2. Staging Distribution: FIGO 2009 vs. FIGO 2023

Stage migration patterns are presented in Table 2 and visualized in Figure S2. Under the FIGO 2009 classification, the majority of patients (81.5%) were categorized as stage I, followed by 5.2% in stage II and 13.3% in stage III. Upon reclassification using the FIGO 2023 criteria, the proportion of stage I disease declined to 65.2%, while stage II rose significantly to 21.9%, largely driven by the newly introduced stage IIC, which alone comprised 14.8% of cases. The proportion of stage III remained relatively stable at 12.9%.

Of the 2969 patients analyzed, 600 (20.2%) experienced stage migration. Upstaging accounted for 98.3% (*n* = 590), all originating from FIGO 2009 stage I. Among those upstaged, 80.8% (477/590) were reclassified to stage IC or IIC based on adverse histologic subtypes or p53 abnormalities, while 19.2% (113/590) were reassigned to stage IIB due to substantial lymphovascular space invasion (LVSI). In contrast, ten patients previously classified as stage IIIA were downstaged under the 2023 system—nine to stage IA3 and one to IIC.

### 3.3. Survival Outcomes According to Stage Migration and Staging Systems

The median follow-up duration was 42.9 months (interquartile range, 14.5–71.2). During this period, 276 patients (9.3%) developed disease recurrence, and 223 (7.5%) died of endometrial cancer. Table 3 summarizes survival outcomes across the FIGO 2009 and FIGO 2023 staging systems. Overall, reclassification under the 2023 criteria led to a modest improvement in outcomes for patients with stage I disease—5-year PFS increased by 3.4%, and OS by 2.8%. The most notable improvement was observed in patients reclassified to stage IB, where 5-year PFS and OS rose by 9.3% and 7.4%, respectively. In contrast, survival rates declined slightly in stage II following restaging (PFS −2.7%, OS −1.5%), with the newly introduced stage IIC subgroup showing the poorest prognosis within this group (PFS: 76.5%, OS: 83.1%). Stage III outcomes remained largely unchanged between the two systems.

**Figure 1:**
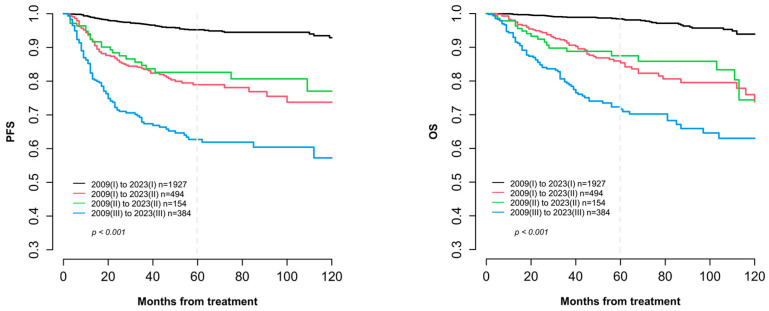
Survival outcomes according to stage migration status following restaging from FIGO 2009 to FIGO 2023. The dash line indicates the 60-month follow-up time point. **Abbreviation:** FIGO, International Federation of Gynecology and Obstetrics; PFS, progression free survival; OS, overall survival.

Survival differences were also examined according to stage migration status. As shown in Figure 1, although survival rates between upstaged and non-upstaged stage II patients appeared similar, survival differences across all groups were statistically significant (*p* < 0.001). Table 3 also details outcomes according to stage migration. Among patients restaged to FIGO 2023 stage I, survival remained excellent across all substages, with 5-year PFS exceeding 89.7% and OS surpassing 95.5%. Notably, all nine patients downstaged to stage IA3 achieved 100% 5-year survival. Conversely, among those upstaged to FIGO 2023 stage II, outcomes were less favorable (Table 4). The 5-year PFS and OS for patients upstaged from stage I to stage II were 79.0% and 85.4%, respectively, which were slightly lower than those of patients consistently staged as FIGO 2009 stage II (PFS: 82.6%, OS: 87.5%). In particular, outcomes for patients upstaged to FIGO 2023 stage IIC varied by their original stage. Those migrating from stage IA to IIC had 5-year PFS and OS of 82.6% and 86.6%, respectively. Patients migrating from stage IB exhibited the poorest outcomes (PFS: 69.2%, OS: 79.7%), while those from stage II to IIC showed further reduction (PFS: 65.0%, OS: 75.1%).

Among patients initially classified as FIGO 2009 stage I, survival outcomes varied considerably according to their reassigned 2023 substages. As shown in Figure S3, 5-year PFS ranged from 69.2% to 96.7%, and 5-year OS from 79.7% to 98.8%, reflecting the prognostic heterogeneity introduced by the updated system.

### 3.4. Survival Analysis by Molecular Classification

Molecular subgroup analysis is presented in Figure S4. No significant difference in 5-year progression-free survival (PFS) was observed between MMR-proficient and MMR-deficient tumors (43.8% vs. 54.6%, *p* = 0.237). Similarly, the 5-year OS rates for MMR-proficient (81.4%) and MMR-deficient (92.8%) groups did not differ significantly (*p* = 0.082). In contrast, tumors with p53 abnormalities were associated with significantly worse outcomes compared to p53-normal tumors, with 5-year PFS rates of 70.8% versus 79.9% (*p* = 0.015) and 5-year OS rates of 76.6% versus 91.7% (*p* < 0.001), respectively. As POLE mutation testing was not routinely performed, survival outcomes for POLE-mutant tumors could not be analyzed separately. Furthermore, the prognostic behavior of tumors falling into the NSMP category remains unclear in this cohort.

### 3.5. Prognostic Performance of FIGO 2023 vs. FIGO 2009 Staging

The prognostic performance of the FIGO 2009 and FIGO 2023 staging systems was compared based on 5-year PFS and OS outcomes. For 5-year PFS, the FIGO 2023 system demonstrated improved model fit with lower AIC (3876.856 vs. 3933.339) and BIC (3920.301 vs. 3955.062) values. A similar pattern was observed for 5-year OS, with lower AIC (2867.131 vs. 2926.834) and BIC (2908.017 vs. 2947.277) values for the 2023 system. The C-index for PFS showed a marginal improvement (0.811 vs. 0.812), whereas the C-index for OS improved more substantially from 0.823 to 0.846, indicating better prognostic discrimination.

**Figure 2:**
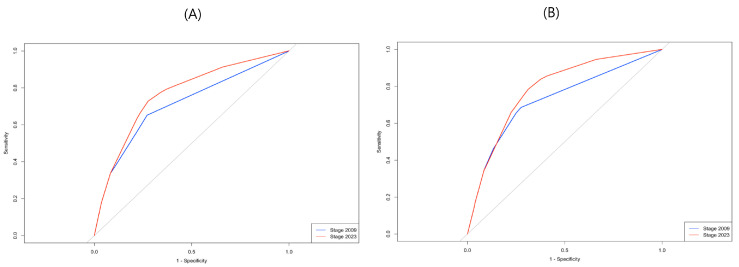
ROC curves comparing the prognostic performance of the FIGO 2009 and FIGO 2023 staging systems for survival prediction. The grey line represents the ROC curve of a random classifier (diagonal line from (0,0) to (1,1)), which serves as the baseline where the true positive rate equals the false positive rate. (**A**) 5-year progression free survival, (**B**) 5-year overall survival. ROC, Receiver operating characteristic; FIGO, International Federation of Gynecology and Obstetrics.

In ROC curve analysis (Figure 2), the FIGO 2023 system demonstrated superior predictive performance, with a higher AUC for both 5-year PFS (0.764 vs. 0.710) and 5-year OS (0.785 vs. 0.728), compared to the FIGO 2009 system.

## 4. Discussion

This study investigated the prognostic impact of the 2023 FIGO staging revision in patients with endometrial cancer, demonstrating substantial stage migration and improved risk stratification compared to the 2009 system. Stage migration occurred in 20.2% of patients, largely due to the integration of aggressive histological subtypes and substantial LVSI into the new staging criteria. Following reclassification, the proportion of stage I tumors decreased from 81.5% to 65.2%, while stage II tumors increased markedly to 21.9%, with a significant proportion allocated to the newly designated stage IIC category.

Survival outcomes closely reflected these shifts. Patients who remained classified as stage I exhibited excellent prognoses, with 5-year PFS and OS rates of 95.2% and 98.4%, respectively. Conversely, patients upstaged to stage II demonstrated survival outcomes comparable to those originally assigned to stage II (5-year PFS: 79.0%, OS: 85.4%). Subgroup analysis further revealed that individuals classified into stages IIB and IIC predominantly exhibited aggressive histologic subtypes or substantial LVSI, consistent with prior studies linking these pathological features to increased recurrence risk and poorer survival outcomes [4,13]. In patients initially staged as FIGO 2009 stage IIIA, downstaging was primarily observed in cases of isolated adnexal metastasis, with these patients achieving a 5-year OS of 100%, supporting earlier findings that adnexal involvement alone does not portend a poor prognosis [14]. Moreover, the adverse prognostic impact of p53 abnormalities observed in this cohort aligns with previous reports demonstrating that p53-abnormal tumors are associated with inferior survival, even among early-stage disease [15,16]. In contrast, MMR status did not significantly affect survival outcomes in this study, mirroring previous research showing inconsistent prognostic implications for MMR deficiency [17,18].

Several recent studies corroborate these findings. Retrospective analyses applying the FIGO 2023 staging system reported stage migration rates between 23.4% and 27.6% in endometrial cancer cohorts [19,20]. Importantly, despite the occurrence of stage reassignment, survival differences between stages became more distinct under the updated system, suggesting that the 2023 revision offers improved prognostic stratification compared to the 2009 framework. A large multi-institutional analysis from Korea and Taiwan reported an 18% upstaging rate, with survival differences becoming clearer under the 2023 criteria [21]. In a European cohort, a 15.5% migration rate was observed, again with improved 5-year PFS among reclassified groups [22]. An international pooled analysis across three ESGO-accredited centers demonstrated stage migration in approximately 25% of cases and confirmed enhanced prognostic precision with the new system [23]. A SEER-based study further validated the prognostic utility of the 2023 classification, showing improved cancer-specific survival discrimination and superiority across AUC, C-index, and decision-curve analyses [24]. Finally, a single-institution retrospective series from India reported significant stage shifts, particularly among early-stage cases, with corresponding differences in 3-year OS and DFS [25]. Collectively, these studies reinforce the consistency of our findings and underscore the broader prognostic robustness of the FIGO 2023 classification.

Beyond changes in stage distribution and survival patterns, the 2023 FIGO staging system demonstrated clear advantages in prognostic discrimination. Quantitative model comparison revealed improved predictive performance of the 2023 system, with consistently lower AIC and BIC values for both progression-free and overall survival. Additionally, the increase in C-index from 0.823 to 0.846 for overall survival indicates a meaningful improvement in the model’s concordance between predicted and observed outcomes. Notably, the AUC for 5-year OS also rose from 0.728 under the 2009 system to 0.785 with the 2023 revision, suggesting enhanced predictive accuracy. These improvements underscore the staging system’s ability not only to reclassify patients more appropriately but also to more reliably distinguish between high- and low-risk groups. In clinical practice, such refinement supports better-informed treatment planning and risk-adapted follow-up strategies.

Notably, the median age at diagnosis in our cohort was 53.7 years, which is concordant with prior Korean population-based studies and cohort analyses reporting mean or median ages of approximately 53–54 years [26,27,28]. Similar trends have been observed in other Asian populations, with a large Chinese cohort reporting a median age at diagnosis of 55 years, which is younger than that typically seen in Western cohorts [29]. In the United States, for example, data from the Surveillance, Epidemiology, and End Results (SEER) program indicate that the median age at diagnosis of uterine cancer is 64 years [30]. Accordingly, our findings are representative of the age distribution observed in real-world Korean patients. When considered by age groups, the results can be generalized, as the prognostic comparison between FIGO 2009 and 2023 staging systems is not inherently age-specific.

The major strength of this study lies in the large, well-characterized cohort, combined with comprehensive clinical and pathological data, which enabled robust external validation of the 2023 FIGO system’s prognostic utility. The use of multiple survival prediction metrics (AIC, BIC, C-index, and AUC) further strengthens the credibility and robustness of these findings. Nonetheless, certain limitations warrant consideration. First, the retrospective nature of the study introduces potential selection biases. Second, incomplete molecular profiling limited the comprehensive stratification of tumors into molecular subgroups. POLE mutation testing would have required NGS and could not be applied retrospectively to archived specimens because of cost and tissue storage constraints. Moreover, p53 or MMR testing were not performed in the majority of patients (81.4%), reflecting the limited availability of molecular assays during much of the study period. As a result, comprehensive re-evaluation of all histological samples was not feasible in this retrospective real-world setting. These constraints reduced the completeness of molecular classification and should be recognized as an inherent limitation of our study. Third, treatment regimens were not standardized across stages, precluding a detailed analysis of survival differences according to adjuvant therapies. As a result, optimal management strategies for patients experiencing stage migration remain to be elucidated. Finally, the long observation period encompassed substantial advances in diagnostic imaging, minimally invasive surgery, radiotherapy techniques, and evolving adjuvant treatment guidelines. These temporal changes may have influenced individual patient outcomes. However, because the primary objective of our study was to evaluate the comparative prognostic performance of the 2009 and 2023 FIGO staging systems, the relative effect of staging reclassification is unlikely to have been materially altered by such practice variations.

## 5. Conclusions

In conclusion, the 2023 FIGO staging system represents a significant advancement in endometrial cancer classification, offering improved prognostic precision by incorporating histological aggressiveness, substantial LVSI, and molecular features. These refinements support the implementation of more individualized, risk-adapted treatment strategies. Future prospective studies, incorporating complete molecular profiling and standardized treatment protocols, will be critical to further validating the utility of the revised staging framework and optimizing patient outcomes.

## Figures and Tables

**Table 1:** Clinicopathological characteristics of patients with endometrial cancer in the study cohort (*n* = 2969) ^1^.

Variables	Patients (*n* = 2969)	%
Age (year)	53.7 ± 10.6	N/A
BMI (kg/m^2^)	25.0 ± 4.5	N/A
Histological type		
Endometrioid	2627	88.5
Serous	107	3.6
Clear cell	58	2.0
Carcinosarcoma	118	4.0
Mesonephric	18	0.6
Mucinous	26	0.9
Poorly differentiated	15	0.5
Size of tumor (mm)	30 ± 24	N/A
Tumor grade		
1	1580	53.2
2	765	25.8
3	473	15.9
Unknown	151	5.1
Myometrial invasion		
Confined to EM	1047	35.3
EM < 1/2	1189	40.0
EM ≥ 1/2	667	22.5
Unknown	66	2.2
LVSI		
Negative	2417	81.4
Focal	172	5.8
Substantial	317	10.7
Unknown	63	2.1
Cervical stromal invasion		
Negative	2600	87.6
Positive	319	10.7
Unknown	50	1.7
Adnexal involvement		
No	2812	94.7
Yes	94	3.2
Unknown	63	2.1
Pelvic cytology		
Negative	2003	67.5
Positive	393	13.2
Unknown	573	19.3
Surgical approach		
MIS	1985	66.8
Laparotomy	929	31.1
Unknown	61	2.1
Adjuvant treatment		
No treatment	1776	59.8
Radiotherapy	938	31.6
Chemotherapy	194	6.5
Others	61	2.1
Type of radiotherapy		
Brachytherapy	387	13.0
EBRT	563	19.0
Not applicable	2019	68.0
MMR status		
Proficient	148	5.0
Deficient	82	2.7
Missing	2739	92.3
P53 status		
Wild type	325	10.9
Mutated	181	6.1
Missing	2463	83.0

^1^ Data are presented as mean ± standard deviation, or number with percentage. **Abbreviations:** BMI, body mass index; EM, endometrium; LVSI, lymphovascular space invasion; MIS, minimally invasive surgery; EBRT, external beam radiation therapy; MMR, mismatch repair.

**Table 2:** Stage distribution and migration from FIGO 2009 to FIGO 2023 in endometrial cancer.

**2009 FIGO**		**2023 FIGO**													
		IA1	IA2	IA3	IB	IC	IIA	IIB	IIC ^1^	IIIA1	IIIA2	IIIB1	IIIC1	IIIC2	Total
	IA	932 (45.3)	713 (34.6)	0	0	96 (4.7)		71 (3.4)	247 (12.0)	0	0	0	0	0	2059 (100)
	IB	0	0	0	186 (51.4)	0		42 (11.6)	134 (37.0)	0	0	0	0	0	362 (100)
	II	0	0	0	0	0	88 (57.1)	10 (6.5)	56 (36.4)	0	0	0	0	0	154 (100)
	IIIA	0	0	9 (11.4)	0	0	0	0	1 (1.3)	58 (73.4)	11 (13.9)	0	0	0	79 (100)
	IIIB	0	0	0	0	0	0	0	0	0	0	20 (100)	0	0	20 (100)
	IIIC1	0	0	0	0	0	0	0	0	0	0	0	169 (100)	0	169 (100)
	IIIC2	0	0	0	0	0	0	0	0	0	0	0	0	126 (100)	126 (100)
	Total	932 (31.4)	713 (24.0)	9 (0.3)	186 (6.3)	96 (3.2)	88 (3.0)	123 (4.1)	438 (14.8)	58 (2.0)	11 (0.4)	20 (0.7)	169 (5.7)	126 (4.2)	2969 (100)

Cross-tabulation of stage classification under the 2009 and 2023 FIGO systems. Each row represents patients originally staged under FIGO 2009 and their corresponding reclassification under FIGO 2023. Values are presented as number of patients, with percentages. Yellow cells indicate patients who remained in the same stage or were subclassified within the same stage. Blue cells represent downstaged patients. Green cells show patients upstaged from IA3 to IIC due to p53 abnormalities. Red cells denote patients upstaged to more advanced stages. ^1^ Under 2009 FIGO staging system, 21 stage IA, 3 stage IB, and 1 stage II patients were upstaged to 2023 FIGO stage IIC due to p53 abnormalities. **Abbreviations:** FIGO, International Federation of Gynecology and Obstetrics.

**Table 3:** Comparison of 5-year progression-free and overall survival according to FIGO 2009 and FIGO 2023 staging systems.

2009 FIGO				2023 FIGO				PFS Difference (%)	OS Difference (%)
Stage	*n* (%)	5-Year PFS Rate in % (95% CI)	5-Year OS Rate in % (95% CI)	Stage	*n* (%)	5-Year PFS Rate in % (95% CI)	5-Year OS Rate in % (95% CI)		
**I**	**2421 (81.5)**	**91.9 (90.6–93.2)**	**95.7 (94.7–96.7)**	**I**	**1936 (65.2)**	**95.3 (94.1–96.5)**	**98.5 (97.8–99.2)**	3.4	2.8
IA	2059 (69.3)	93.8 (92.5–95.1)	97.0 (96.1–97.9)	IA1	932 (31.4)	96.7 (95.3–98.1)	98.8 (97.9–99.7)		
				IA2	713 (24.0)	94.8 (92.7–96.9)	98.8 (97.9–99.7)		
				IA3	9 (0.3)	100	100		
IB	362 (12.2)	80.4 (75.4–85.4)	88.1 (83.6–92.6)	IB	186 (6.3)	89.7 (83.7–95.7)	95.5 (91.0–100.0)	9.3	7.4
				IC	96 (3.2)	94.2 (88.6–99.8)	96.7 (92.3–100.0)		
**II**	**154 (5.2)**	**82.6 (75.8–89.4)**	**87.5 (81.5–93.5)**	**II**	**649 (21.9)**	**79.9 (76.2–83.6)**	**86.0 (82.7–89.3)**	−2.7	−1.5
				IIA	88 (3.0)	92.1 (85.4–98.8)	93.8 (87.7–99.9)		
				IIB	123 (4.1)	82.5 (74.5–90.5)	90.1 (83.4–96.8)		
				IIC	438 (14.8)	76.5 (71.7–81.3)	83.1 (78.7–87.5)		
**III**	**394 (13.3)**	**63.5 (57.5–69.5)**	**72.2 (66.6–77.8)**	**III**	**384 (12.9)**	**62.7 (56.7–68.7)**	**71.6 (65.9–77.3)**	−0.8	−0.6
IIIA	79 (2.7)	79.5 (68.2–90.8)	86.5 (76.7–96.3)	IIIA1	58 (2.0)	80.2 (68.5–91.9)	84.9 (72.9–96.9)		
				IIIA2	11 (0.4)	62.5 (20.7–100.0)	83.3 (53.5–100.0)		
IIIB	20 (0.7)	42.3 (12.6–72.0)	51.6 (21.2–82.0)	IIIB1	20 (0.7)	42.3 (12.6–72.0)	51.6 (21.2–82.0)		
				IIIB2	0 (0.0)				
IIIC1	169 (5.7)	66.3 (57.7–74.9)	73.8 (65.6–82.0)	IIIC1	169 (5.7)	66.3 (57.7–74.9)	73.8 (65.6–82.0)	0	0
IIIC2	126 (4.2)	53.2 (42.1–64.3)	64.5 (53.8–75.2)	IIIC2	126 (4.2)	53.2 (42.1–64.3)	64.5 (53.8–75.2)	0	0

Five-year PFS and OS rates are compared between the two staging systems across equivalent stages. Differences in survival reflect the prognostic impact of restaging based on the updated FIGO 2023 criteria. **Abbreviations:** FIGO, International Federation of Gynecology and Obstetrics; PFS, progression free survival; OS, overall survival.

**Table 4:** Stage migration and 5-year survival of patients restaged to FIGO 2023 Stage II.

2009 FIGO → 2023 FIGO	No. of Patients	5-Year PFS Rate in % (95% CI)	5-Year OS Rate in % (95% CI)
I → II	494	79 (74.6–83.4)	85.4 (81.4–89.4)
II → II	154	82.6 (75.8–89.4)	87.5 (81.5–93.5)
II → IIA	88	92.1 (85.4–98.8)	93.8 (87.7–99.9)
IA → IIB	71	84.5 (73.5–95.5)	91.6 (83.6–99.6)
IB → IIB	42	77.3 (63.3–91.3)	85.5 (71.9–99.1)
II → IIB	10	88.9 (68.4–100.0)	100
IA → IIC	247	82.6 (76.5–88.7)	86.6 (81.1–92.1)
IB → IIC	134	69.2 (59.8–78.6)	79.7 (71.1–88.3)
II → IIC	56	65.0 (50.5–79.5)	75.1 (62.2–88.0)

**Abbreviations:** FIGO, International Federation of Gynecology and Obstetrics; PFS, progression free survival; OS, overall survival.

## Data Availability

The data that support the findings of this study are available from the corresponding author upon reasonable request.

## Supplementary Materials

The following supporting information can be downloaded at: https://www.mdpi.com/article/10.3390/cancers17172871/s1, Figure S1. Flow diagram of patient selection for the final analysis. Figure S2. The Sankey diagram depicting stage migration of endometrial cancer patients from FIGO 2009 to FIGO 2023 staging system. Figure S3. Survival outcomes according to stage migration among patients with FIGO 2009 stage I endometrial cancer. Figure S4. Survival outcomes by MMR and p53 status in endometrial cancer patients.

## Abbreviations

The following abbreviations are used in this manuscript:

AIC	Akaike information criterion
AUC	Area under the receiver operating characteristic curve
BIC	Bayesian information criterion
BMI	Body mass index
C-index	Concordance index
EBRT	External beam radiation therapy
EC	Endometrial cancer
EM	Endometrium
FIGO	International Federation of Gynecology and Obstetrics
LVSI	Lymphovascular space invasion
MIS	Minimally invasive surgery
MMR	Mismatch repair
NSMP	No specific molecular profile
OS	Overall survival
PFS	Progression free survival
ROC	Receiver operating characteristic
