# Supplementary material for: Improved Prognostic Stratification with the FIGO 2023 Staging System in Endometrial Cancer: Real-World Validation in 2969 Patients
Source: Cancers (Basel). 2025 Sep 1;17(17):2871. doi: 10.3390/cancers17172871 (PMC12427876; doi:10.3390/cancers17172871)
Supplement: Supplementary file 1 [file cancers-17-02871-s001.zip › cancers-3829283-supplementary.pdf]

Supplementary Materials:

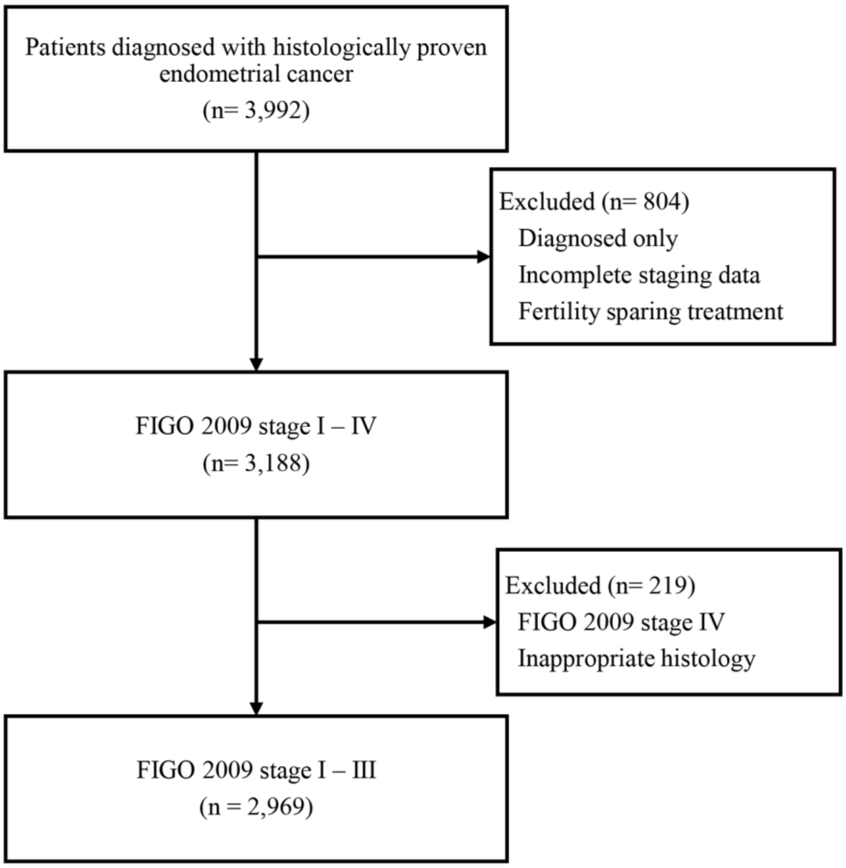

Figure S1. Flow diagram of patient selection for the final analysis.

Abbreviations: FIGO, International Federation of Gynecology and Obstetrics.

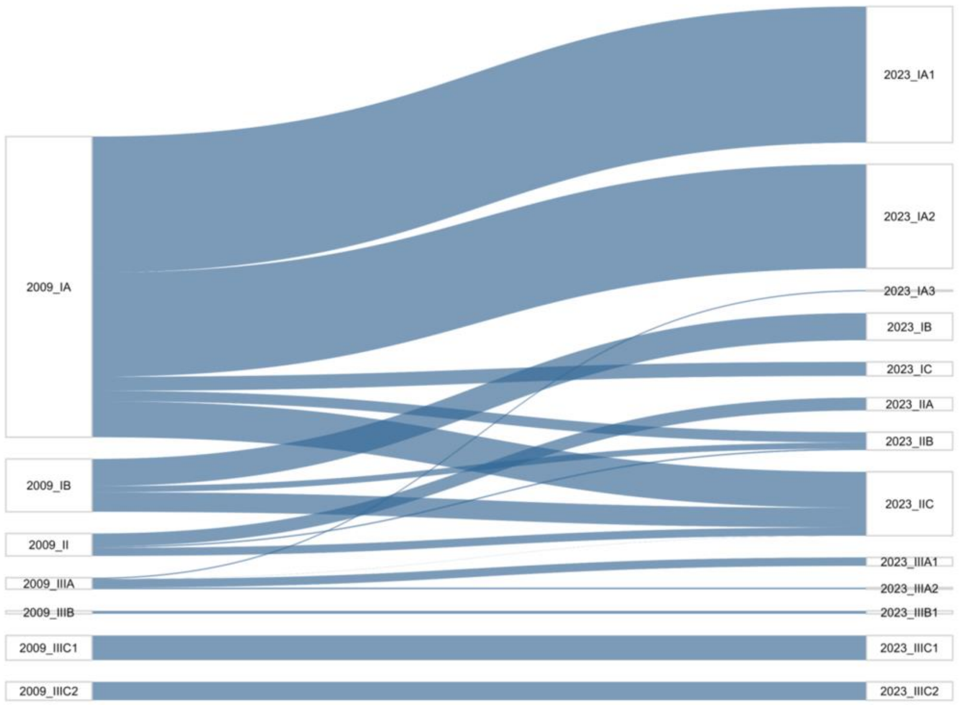

Figure S2. The Sankey diagram depicting stage migration of endometrial cancer patients from FIGO 2009 to FIGO 2023 staging system.

Abbreviations: FIGO, International Federation of Gynecology and Obstetrics.

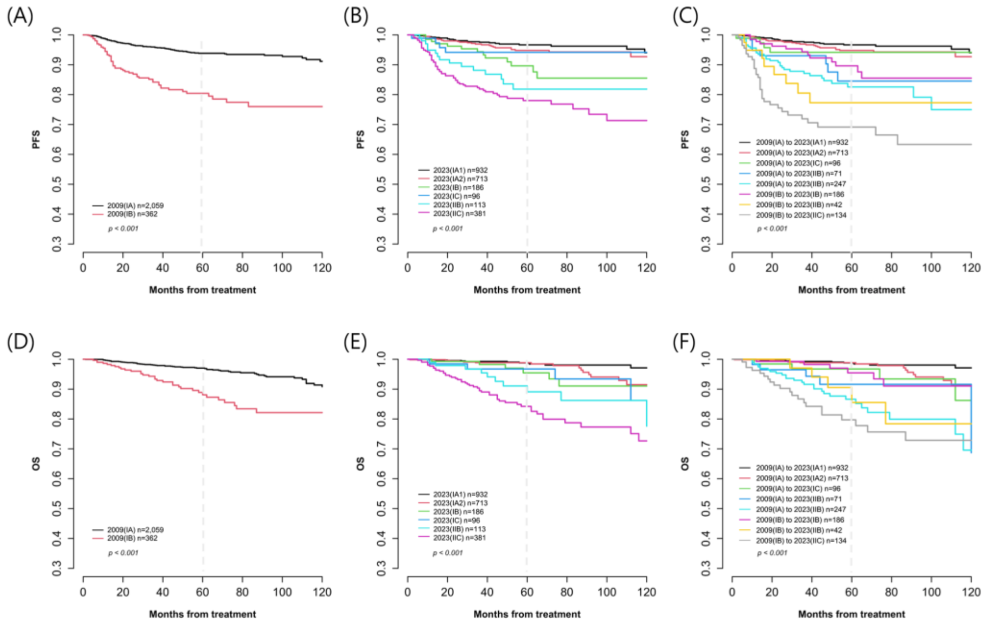

Figure S3. Survival outcomes according to stage migration among patients with FIGO 2009 stage I endometrial cancer.

(A) Kaplan–Meier curves for PFS in patients with FIGO 2009 stage I, (B) PFS according to FIGO 2023 stages in patients initially classified as FIGO 2009 stage

I, (C) PFS stratified by stage migration patterns from FIGO 2009 stage I to corresponding FIGO 2023 subgroups, (D) Kaplan–Meier curves for OS in patients with FIGO 2009 stage I, (E) OS according to FIGO 2023 stages in patients initially classified as FIGO 2009 stage I, (F) OS stratified by stage migration patterns from FIGO 2009 stage I to corresponding FIGO 2023 subgroups.

Abbreviations: FIGO, International Federation of Gynecology and Obstetrics; PFS, progression free survival; OS, overall survival

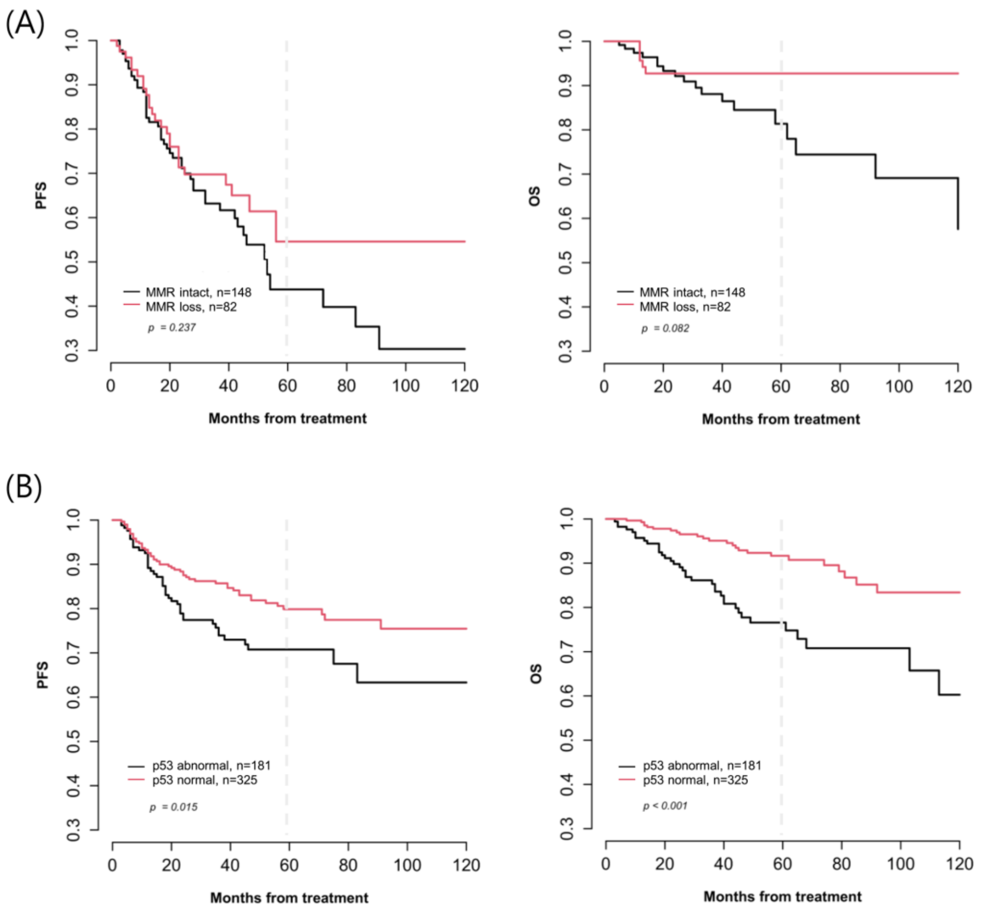

Figure S4. Survival outcomes by MMR and p53 status in endometrial cancer patients.

(A) MMR status, (B) By p53 status.

Abbreviations: MMR, mismatch repair; PFS, progression free survival; OS, overall survival
